# Differences in metabonomic profiles of abdominal subcutaneous adipose tissue in women with polycystic ovary syndrome

**DOI:** 10.3389/fendo.2023.1077604

**Published:** 2023-02-24

**Authors:** Fangfang Di, Danfeng Gao, Lihua Yao, Runjie Zhang, Jin Qiu, Liwen Song

**Affiliations:** ^1^ Obstetrics and Gynecology Department, Tongren Hospital, Shanghai Jiao Tong University School of Medicine, Shanghai, China; ^2^ Hongqiao International Institute of Medicine, Tongren Hospital, Shanghai Jiao Tong University School of Medicine, Shanghai, China

**Keywords:** polycystic ovary syndrome, metabonomics, proliferation, apoptosis, mitochondrial function

## Abstract

**Introduction:**

Polycystic ovary syndrome (PCOS) is a complex endocrine disorder that often coexists with a metabolic disorder. Studies have demonstrated that the malfunction of adipose tissue, particularly abdominal adipose tissue, could exacerbate reproductive and metabolic problems in PCOS patients. Adipose tissue-secreted signaling mediators (e.g., lipids and metabolites) would then interact with other body organs, including the ovary, to maintain the systemic equilibrium.

**Methods:**

In this study, we examined adipose samples from PCOS patients and unaffected individuals using a liquid chromatography–mass spectrometry-based metabonomics approach (LC–MS/MS). PCOS biomarkers were selected using multivariate statistical analysis.

**Results:**

Our pathway analysis revealed that these differential metabolites could be engaged in inflammatory diseases and mitochondrial beta-oxidation. We further developed an in vitro PCOS cell model to examine the effects of hyperandrogenism on granulosa cells and related metabolic disorders. We noted that isoleucine recovered the promotive effect on cell apoptosis, inhibitory effect on cell proliferation, sex hormone secretion, and mitochondrial function induced by dehydroepiandrosterone. Our gas chromatography–mass spectrometry targeted analysis (GC–MS/MS) revealed that isoleucine was significantly decreased in PCOS patients.

**Discussion:**

Based on these results, we speculate that metabolome alterations are vital in ameliorating PCOS symptoms. This may be a novel therapeutic target for PCOS treatment. Our study provides preliminary evidence that these findings will enhance our ability to accurately diagnose and intervene in PCOS.

## PRÉCIS

Isoleucine demonstrated a high PCOS diagnostic ability and recovered promotive effect on cell apoptosis, inhibitory effect on cell proliferation, sex hormone secretion, and mitochondrial function induced by dehydroepiandrosterone in granulosa cells.

## Introduction

Polycystic ovary syndrome (PCOS) is a complex endocrinopathy in women, affecting approximately 6% to 21% of women of reproductive age (1–3). It has been extensively investigated as it is considered the most prevalent form of ovarian disorder. PCOS has been identified as a multisystem disease and is no longer regarded merely as a disease of ovary. In addition to having a wide range of symptoms and indicators that make categorizing the illness challenging, patients with PCOS are also linked to a number of other conditions, such as menstrual irregularities and metabolic changes (4). Therefore, the etiology of PCOS is not fully understood. Currently, suppressive or maintenance treatment including metformin, hormonal contraceptives, and orlistat are the first-line treatment for PCOS (5, 6). The efficacy of these treatments has been limited by low sustainability and adherence. Therefore, it is necessary to search for new replacements to provide ideal treatment options for PCOS patients.

Adiposity is one of the main symptoms of PCOS and is believed to be the main cause of increased metabolic risk (7). Adipose tissue (AT) has significant endocrine and metabolic functions as is known as an organ for storing fat (8). Extensive research has been done on the functions of subcutaneous AT in PCOS women (9, 10). Beyond proteins, adipocytokines—which are released by AT—also involve lipids, extracellular vesicles, metabolites, and noncoding RNAs in the process whereby AT communicates with other body organs (11). Particularly, mounting evidence indicates that adiponectin, an adipocytokine, has a critical role in female reproductive organs, where it promotes IGF-I-induced steroidogenesis in granulosa cells (12). Although these adipose tissue secretions are essential to PCOS, a thorough explanation of the potential mechanisms is required. Therefore, identifying the adipokines, including metabolites secreted by AT and the characterization of their effects on PCOS, is of utmost importance.

It is necessary to identify prospective biomarkers by adopting non-invasive methods and precise techniques to gain a better understanding of the pathomechanisms of PCOS. Metabonomics, a recently developed technology, describes dynamic changes and provides information for the discovery of active drivers on the metabolites present in physiological or pathological systems (13). It has been widely applied in the diagnosis of a variety of disorders (14). Recently, a research group analyzed several metabolomic studies concerning PCOS-affected women and contrasted them with data from healthy controls (15, 16). Certain metabolites, such as fatty acids (17) and amino acids (10), have been recognized as biomarkers for the diagnosis of PCOS. It is possible to trace miniscule biochemical alterations in this endocrinopathy by employing metabonomics to investigate the pathophysiology of PCOS, which may aid in the diagnosis of the disorder. Uncertainty surrounds the function of the active metabolites released by AT in PCOS.

To offer fresh perspectives on the part AT plays in the pathophysiology of PCOS, we conducted non-targeted metabonomics using biopsies obtained during surgery in women with or without PCOS. In contrast to conventional molecular genetic approaches, our current experimental methodology offers the theoretical benefit of a hypothesis-free technique to identify the dysregulated metabolites in AT of PCOS patients. In addition, we investigated the protective effect of metabolites on proliferation, apoptosis, and mitochondrial function in dehydroepiandrosterone (DHEA)-induced PCOS cell models. Our findings made clear the function of released metabolites related with AT in PCOS. The results of the present research offer a novel perspective on the potential therapeutic benefits and molecular activities in PCOS.

## Materials and methods

### Recruitment of patients

A total of 16 women were enrolled in this study. Eight women suffered from PCOS, while the remaining eight were normal control (NC) women. The subjects’ average age and BMI were statistically identical (**Table 1**). The study was approved by the ethics committee of Shanghai Tongren Hospital, Shanghai Jiao Tong University School of Medicine. All patients provided informed consent. The Rotterdam criteria (18), which include polycystic ovaries, oligo- or anovulation, clinical and/or hyperandrogenism, and exclusion of other causes of hyperandrogenism like androgen-secreting tumors, non-classical congenital adrenal hyperplasia, hyperprolactinemia, and Cushing’s syndrome, were used to diagnose PCOS. When two or more of the three requirements were met, the diagnosis was deemed accurate. The women in the non-PCOS group had regular menstrual cycles every 26–34 days with no signs of hyperandrogenism. Additionally, none of the study participants had used any medications known to impact metabolic or hormonal characteristics within the 3 months prior to the study.

**Table 1:** Biochemical indexes from controls and PCOS patients.

	Control (n = 8)	PCOS (n = 8)
Age (years)	29.75 ± 3.37	28.6 ± 2.97
BMI (kg/m^2^)	20.53 ± 2.71	21.14 ± 4.48
Basal FSH (IU/L)	5.67 ± 2.23	6.58 ± 1.62
Basal LH (IU/L)	6.79 ± 2.56	14.28 ± 4.32*
Basal T_0_ (nmol/mL)	1.64 ± 0.38	3.04 ± 1.05*

T_0_, testosterone.

Data are presented as mean ± SEM.

*P < 0.05 versus the control group.

### Sample preparation

Subcutaneous fat of the abdomen (PCOS, 5; NC, 8) was sampled from women with tubal factor infertility using laparoscopy. Three patients dropped out of the trial. When performing the laparoscopy, all patients were fasting, and the surgeon removed 3–4 g of subcutaneous fat. Biopsies were immediately rinsed in cooled NaCl 0.9% solution, segmented, and snap-frozen as previously reported (9, 18). Until analysis, samples were kept frozen in liquid nitrogen (196°C).

Around 20 mg of subcutaneous fat of the abdomen was added to 500 μl methanol solution (containing 5 μg/ml L-2-chlorophenylalanine as internal standard) and homogenized for 2 min. The supernatant was centrifuged at 13,000 rpm at 4°C for 10 min, and 200 μl was obtained. The same volume of serum was extracted from all samples and mixed evenly to prepare QC (quality control) samples.

### LC–MS/MS analysis

The UHPLC system was coupled to Orbitrap/MS (Waters Corp., Milford, MA, USA), equipped with an electrospray ionization source, and operated in positive or negative ionization modes with a mass resolution of 70,000 and an m/z of 200. Using data correlation (dd-MS2, TopN = 10) MS/MS mode, the full-scan quality resolution was 17,500 when m/z was 200, and the scanning range was 100–1,500. The chromatographic conditions were as follows: sample size was 2 μl, column temperature was 25°C, flow rate was 0.35 ml/min, and mobile phases were 0.1% formic acid aqueous solution and 0.1% acetonitrile formic acid solution. The optimized chromatographic gradient was as follows: 0–2 min, 5% in liquid B; 2–10 min, 5%–95% in liquid B; 10–15 min, 95% in liquid B; 15–18 min, 5% in liquid B. Data were obtained in centroid mode using Thermo Excalibur 2.2 software from Thermo Fisher Scientific, Massachusetts, USA.

### GC–MS/MS analysis

Serum sample collection was referred to Yan et al. (19). Shanghai Lu-Ming Biotech Company Limited (Shanghai, China) provided an experimental platform and assistance for the targeting amino acid metabolomics analysis. Briefly, a mixture of methanol/water (4:1 by volume) was used to collect 2 × 10^7^ per sample. The sample was quickly stored in liquid nitrogen. Before testing on the machine, the sample was equilibrated to ambient temperature for 30 min. The sample was dispersed using the ultrasonic lysis method. It was then concentrated and centrifuged and then freeze-dried. Finally, a mixture of BSTFA and n-hexane (4:1 by volume) was added to the sample, vortexed vigorously for 2 min, and derivatized at 70°C for 60 min. These samples were analyzed by a gas chromatography system (Thermo Fisher Scientific TSQ 9000, USA). UPLC–ESI–MS/MS was utilized as the analytical method for the quantitative detection of targeted amino acid metabolites.

### Cell line culture

A steroid-derived human granulosa-like tumor cell line (KGN cell line) was selected for this study because it retains the physiological properties of ovarian granulosa cells (20). In the DMEM/F-12 medium including 10% fetal bovine serum (FBS, Gibco), KGN cells were grown in a humid atmosphere at 37°C and 5% CO_2_. The concentrations of metabolites used are listed in the legend. DHEA was added to the medium 4 h prior to metabolite treatment.

### Proliferation assays

The effects of metabolites were assessed using the Cell Counting Kit-8 assay (CCK-8, Beyotime, China). KGN cells were reseeded in 96-well plates at 5,000 cells/well. Each well received 10 l of CCK-8 reagent. Cells were then incubated for 1 h at 37°C. Absorbance was measured at a wavelength of 450 nm using a microplate reader (Thermo Fisher Scientific, USA). Cells were assessed at 0, 24, and 48 h.

### Apoptosis analysis

A flow cytometry study of the Annexin V-fluorescein isothiocyanate (FITC) against propidium iodide (PI) assay was used to identify apoptosis (BD Pharmingen, CA, USA). A total of 3 × 10^5^ KGN cells were reseeded in each well of six-well plates. Cells were trypsinized, washed twice with cold phosphate-buffered saline for 15 min at room temperature, and used to incubate cells with 5 µl of Annexin v-FITC and 5 µl of PI each well.

### Measurement of hormones

Serum samples extracted from KGN culture supernatants were tested in duplicate using testosterone (T_0_), estrogen (E_2_), and progesterone (P_4_) ELISA kits (CUSABIO, China) in accordance with the directions provided by the manufacturer.

### Measurement of ATP contents

Using the appropriate kits, adenosine triphosphate (ATP) concentrations were assessed following the extraction and quantification of proteins from KGN (Beyotime, China).

### Statistical analysis

Data were obtained using the Thermo Xcalibur 2.2 software (Thermo Scientific, SAN Jose, USA). Peak calibration and extraction were performed using the Compound Discovery Software (Thermo Fisher Scientific). Data tables were imported into Simca-P 13.0 for multivariate statistical analysis. Unsupervised principal component analysis (PCA) was used to assess the overall trend of separation between these samples. Differential metabolites were screened using partial least square, discrimination analysis (PLSDA). According to the PLSDA model, variables whose importance was greater than 1.0 in the projection (VIP) value were selected, and SPSS Statistics 18.0.0 was used for the two-tailed Student’s t test, and a p-value < 0.05 was considered statistically significant. Multiple test adjustments were made using Bonferroni correction. To identify these potential biomarkers, the accurate ion mass was entered into the Human Metabolome Database (HMDB), Metlin, MoNA, and MassBank databases to match accurate molecular weight and automatically search for MS1/MS2 fragment ions. Finally, to determine the structure of the compound, we used our internal standard metabolite library, matching the exact mass, fragment ion mass, and retention time. Furthermore, metabolic pathway enrichment analysis was performed using the KEGG database (https://www.metaboanalyst.ca/). The area under the receiver operating characteristic (ROC) curve, i.e., AUC, was used to evaluate the diagnostic ability of biomarkers. The metabolite interaction network analysis was conducted using the IPA online database.

## Results

### Identification and screening of differentially expressed metabolites

**Figure 1:**
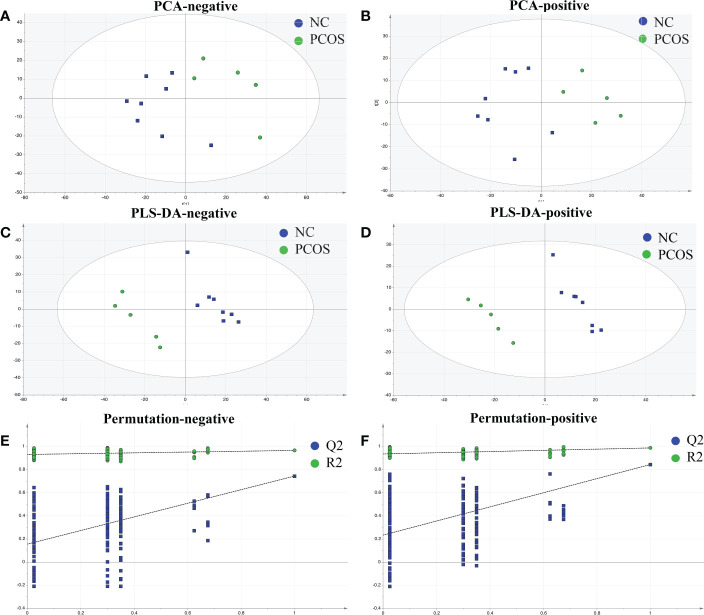
Identified metabolic profile in NC and PCOS. **(A, B)** PCA revealed a distinct metabolic profile in the PCOS group compared with the NC group. The X-axis and Y-axis represent the first and second principal components, respectively. **(C, D)** Statistical validation with perripening analysis of the corresponding OPLS–DA model of the NC and PCOS groups. **(E, F)** Perripening tests were obtained from LC–MS data of the NC and PCOS groups. The intercept values of the regression line and the Y-axis are R2 and Q2.

Patients’ AT was drawn for metabonomics analysis to examine the metabolic profiles of the NC and PCOS groups. The findings of the PCA for the NC and PCOS groups (**Figures 1A, B**) revealed a pattern of aggregation within groups and segregation between groups. The plots of the PCOS group were very different from those of the NC group, according to the PLS-DA model, which revealed significant metabolite differences between the two groups (**Figures 1C, D**). Additionally, a continuing analysis of the PLS-DA model was carried out (**Figures 1E, F**). In the PCOS group, a total of 107 distinct metabolites were found (VIP >1, p < 0.05).

### Enrichment analysis on differential metabolites

**Figure 2:**
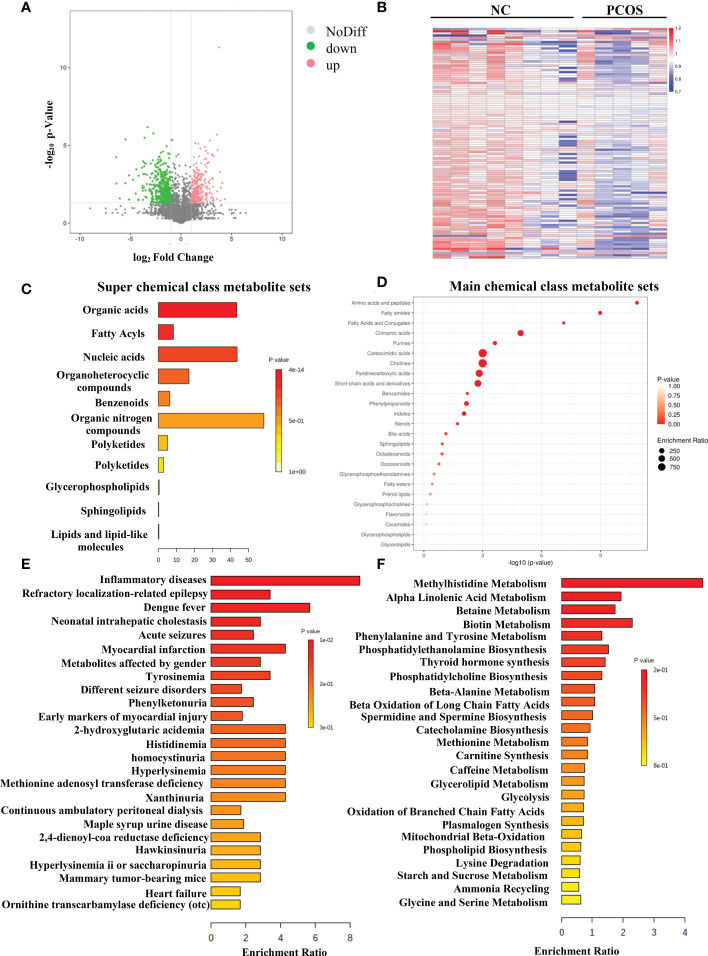
Differentially expressed metabolites in PCOS. **(A)** The volcano map of all metabolites expressed in NC and PCOS groups. **(B)** Hierarchical clustering analysis was used to assess significantly regulated metabolites between NC and PCOS groups. Increased and decreased metabolites are depicted by red and blue, respectively. **(C)** Super chemical class metabolite sets of the differential metabolites. **(D)** Main chemical class metabolite sets of the differential metabolites. **(E)** Enriched diseases clustering analysis. **(F)** Enriched metabolic pathway clustering analysis.

**Figure 2A** illustrates the results as a volcano plot of all metabolites. Red and green colors were used to represent elevated and decreased metabolites, respectively. **Figure 2B** depicts a heat map of the various metabolites identified by LC–MS. The differential metabolites could be loosely divided into various super-classes as per the enriched metabolite set clustering analysis (**Figure 2C**), such as organic acids, fatty acyls, nucleic acids, and main classes such as amino acids and peptides (**Figure 2D**). The biomarker pathway enrichment analysis used the KEGG and MetaboAnalyst databases. According to **Figures 2E, F**, these differential metabolites were mainly enriched in inflammatory diseases, beta-oxidation, mitochondrial beta-oxidation, etc. It is well known that PCOS is an inflammatory, systemic, lifestyle endocrinopathy (21). The mitochondrion is a vital organelle that regulates energy production necessary for cellular survival, and mitochondrial malfunction has been implicated as a possible pathogenesis-inducing factor for PCOS, according to a previous study (22–24). Consequently, the experiments confirmed that the altered metabolites might play important functions in PCOS.

### The possible function of metabolites

**Figure 3:**
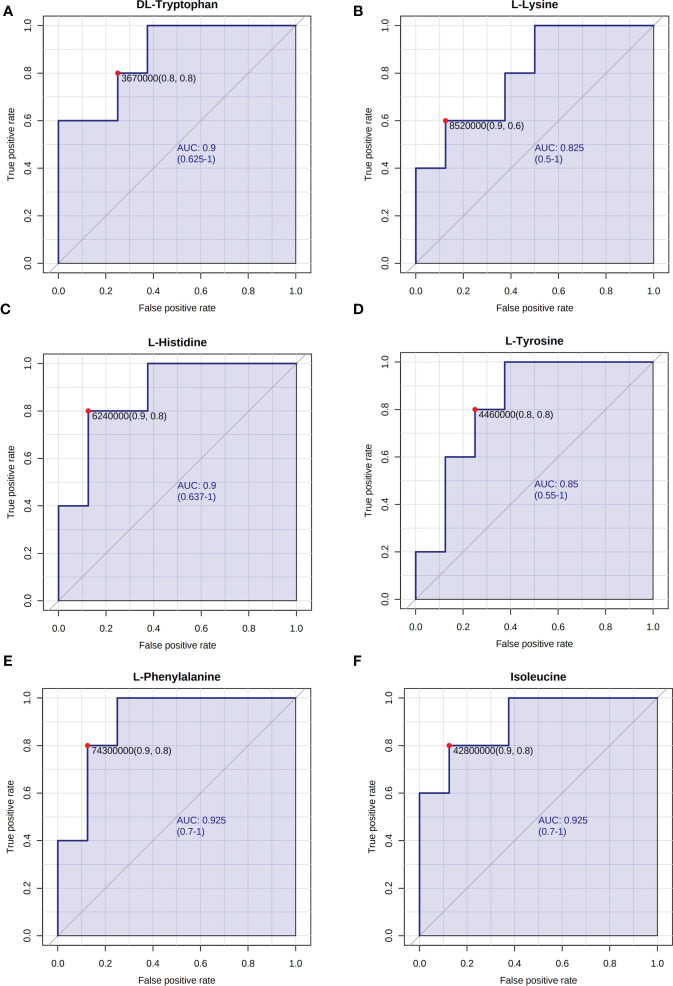
ROC analysis of representative differentially expressed metabolites. The AUC of **(A)** DL-tryptophan, **(B)** L-lysine, **(C)** L-histidine, **(D)** L-tyrosine, **(E)** L-phenylalanine, and **(F)** isoleucine.

**Figure 4:**
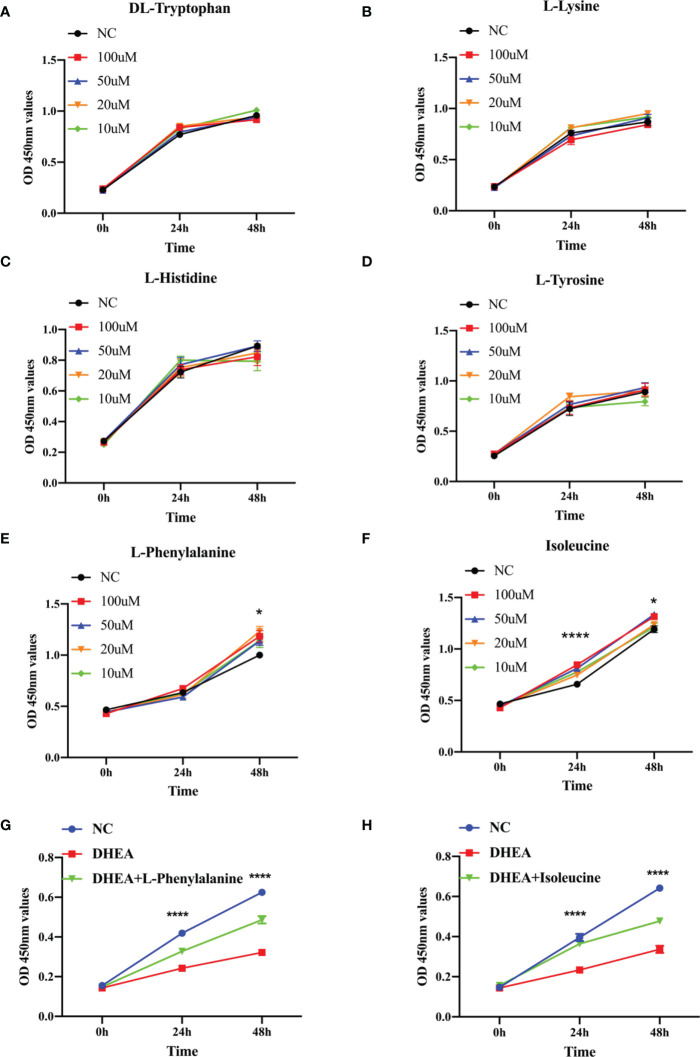
The growth function of representative metabolites in granulosa cells. **(A–F)** CCK-8 assays were used to detect cell viability of KGN cells at different times using the same concentration of metabolites (10, 20, 50, or 100 μM). **(G)**. Relative cell growth after treatment with DHEA (10^−4^ M) and L-phenylalanine (20 μM) was detected by the CCK-8 assay. **(H)**. Relative cell growth after treatment with DHEA (10^−4^ M) and isoleucine (20 μM) was detected by the CCK-8 assay. *p < 0.05, ****p < 0.0001.

Research has shown that granulosa cell death may be the fundamental cause of follicular atresia. Granulosa cell proliferation and apoptosis determine the fate of a follicle (25). The levels of plasma amino acids in PCOS women have been demonstrated to be significantly out of balance (26), given that L-tyrosine and L-leucine may recover regular menstrual cycle and ovulation in PCOS (27). Hence, we chose amino acids such as DL-tryptophan, L-lysine, L-histidine, L-tyrosine, L-phenylalanine, and isoleucine as candidate metabolites to explore their potential biological roles. The ROC analysis uncovered that DL-tryptophan, L-lysine, L-histidine, L-tyrosine, L-phenylalanine, and isoleucine demonstrated a strong capacity for PCOS diagnosis, with AUC values of 0.900, 0.825, 0.900, 0.850, 0.925, and 0.925, respectively (**Figures 3A–F**). This suggested that the amino acids may assist in making a clinical diagnosis. Further studies need to be conducted to confirm the functions of the amino acids. The primary mechanism of follicular atresia in PCOS was found to be increase in granulosa cell apoptosis (28). Cell viability assay (**Figures 4A–F**) was performed, which revealed that L-phenylalanine and isoleucine could significantly promote the proliferation of cells in cultured KGN (**Figures 4E, F**), and the two amino acids were chosen for an in-depth study.

### The effect of L-phenylalanine and isoleucine on KGN cell proliferation and apoptosis in the DHEA cell model

**Figure 5:**
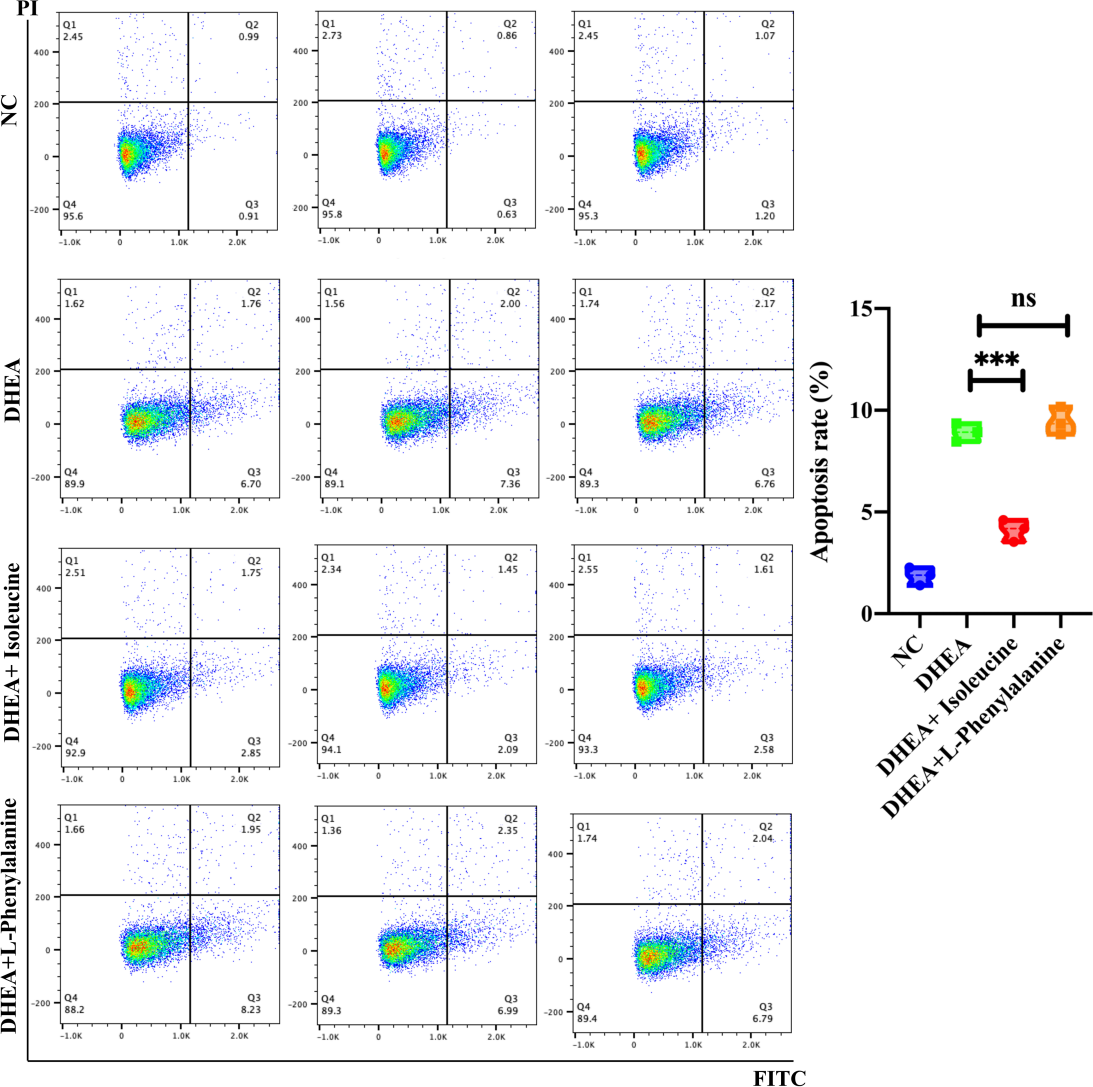
Analysis of the cell apoptosis of KGN cells transfected with DHEA (10^−4^ M) and isoleucine (20 μM) or L-phenylalanine (20 μM). The early and late apoptosis rates of KGN cells were compared using PI and FITC. ***p < 0.001; ns, non-significant.

KGN cells were pretreated with DHEA to imitate the pathophysiological state of PCOS and simulate the hyperandrogenic milieu in order to further investigate the physiological roles of the two amino acids (29). L-Phenylalanine (**Figure 4G**) and isoleucine (**Figure 4H**) significantly reversed the growth inhibition of DHEA-treated KGN cells. To delve deeper into this finding, apoptosis of KGN cells was discovered using flow cytometry. Isoleucine significantly recovered the apoptosis of DHEA-treated KGN cells but not in the L-phenylalanine group (**Figure 5**).

### Isoleucine protected mitochondrial functions and sexual hormone disturbances of KGN after exposure to DHEA

**Figure 6:**
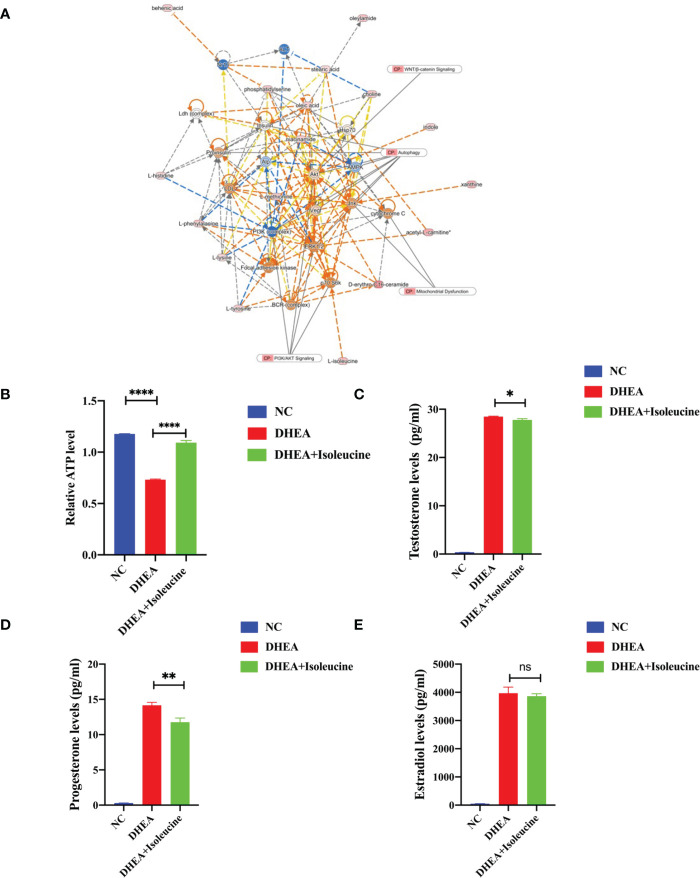
The possible mechanism of isoleucine in granulosa cells. **(A)** Network and function analysis of differential metabolites using the IPA database. The yellow nodes represent the upregulated metabolites. The blue nodes represent the downregulated metabolites. CP represents the signaling pathway related to the changed metabolites. **(B)** Relative ATP level of KGN cells transfected with DHEA (10^−4^ M) and isoleucine (20 μM). Cell serum was collected for ELISA analysis of **(C)** T_0_, **(D)** P_4_, and **(E)** E_2_. *p < 0.05, **p < 0.01, ****p < 0.0001.

**Figure 7:**
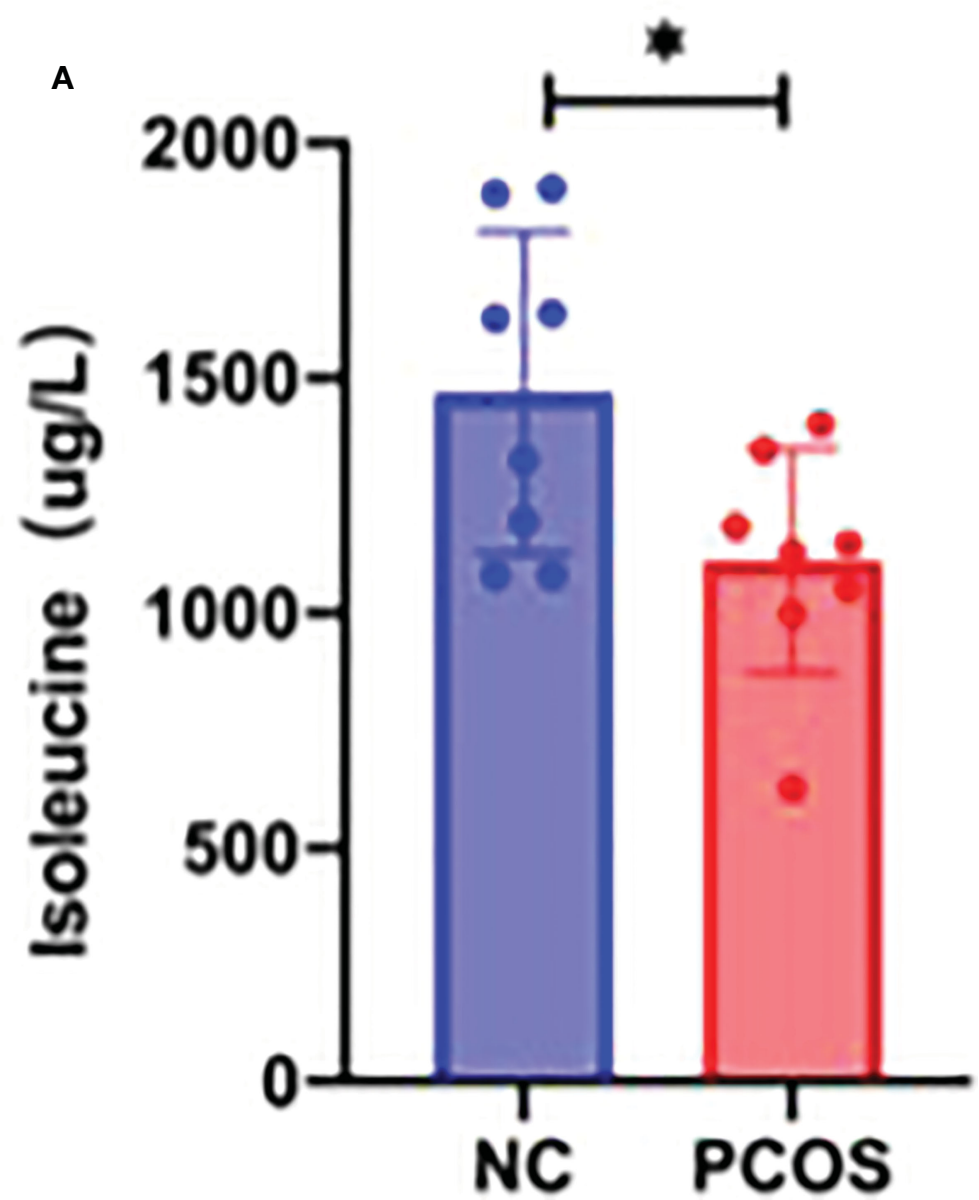
GC–MS/MS-targeted amino acid metabolism analysis. **(A)** Histogram showing the concentration of isoleucine in the NC group and PCOS group. *p < 0.05.

A creative pathway analysis was performed on various metabolites (IPA), which identified numerous linked signaling pathways, including the ones that have been shown to be closely related to PCOS such as pathways related to mitochondrial dysfunction, ROS, PI3K/AKT, MAPK, and Wnt signaling (**Figure 6A**). The mitochondrion is a crucial organelle that controls energy production required for cellular survival. According to earlier reports (22), PCOS pathogenesis could be exacerbated by mitochondrial dysfunction (23, 24). After DHEA treatment, mitochondrial function indicator ATP level decreased markedly (**Figure 6B**), indicating the possibility of mitochondrial malfunction. Isoleucine replenishment reduced the DHEA-induced mitochondrial dysfunction (**Figure 6B**). Additionally, ELISA was used to measure the T_0_, E_2_, and P_4_ in each group. Compared with the control group, the DHEA group had higher T_0_, E_2_, and P_4_ concentrations. However, compared with the DHEA group, the levels of T_0_ and P_4_ but not E_2_ were suppressed in the DHEA+ isoleucine group (**Figures 6C–E**). Consistently, we deduced that DHEA could lead to mitochondrial dysfunction and sexual hormone disturbances in granulosa cells, which were ameliorated by isoleucine treatment. To further clarify the role of isoleucine in PCOS, we detected the levels of intracellular amino acids by GC–MS/MS-targeted amino acid metabolism analysis. Notably, the reduced level of isoleucine could be significantly detected in PCOS (**Figure 7A**). Taken together, these results indicated that isoleucine was the potential target for PCOS treatment.

## Discussion

The primary defect in PCOS appears to be an exaggerated androgen secretion by ovarian theca cells (30) and possibly by the adrenals, upon which several factors act, triggering the development of the PCOS phenotype. Among these factors, abdominal adiposity and/or obesity play a major role in many PCOS patients, in part because of the induction of insulin resistance and hyperinsulinemia. Furthermore, hyperinsulinemia facilitates androgen secretion in the ovaries and adrenals (31). Abdominal AT has been identified as a better marker of metabolic health than body weight (32), and mouse models of PCOS suggest that AT plays a crucial role in the development of PCOS (33). Most metabonomic studies comparing PCOS and normal control subjects have focused on analyses of plasma/serum or follicular fluid (34). Secretion of metabolites from brown adipose tissue (BAT) has recently been studied by our research group in an untargeted manner (25). We were interested in analyzing active metabolites in subcutaneous AT that may influence the PCOS phenotype. Possible reasons include the following: 60% of PCOS-afflicted women are overweight or obese (7), and the bulk of these patients are classified as having abdominal obesity. A recent prevailing view has proven that hyperandrogenism favors accumulation of abdominal fat, thereby promoting insulin resistance (35–37), both of which are diagnostic indicators of PCOS. Thus, the AT is more relevant with PCOS. AT has been employed in prior research on the alteration of gene and protein expressions in PCOS (10, 31, 38). To date, few studies have focused on the metabolic function of AT in paracrine. Thus, we employed the abdominal AT to investigate the connection of released metabolites and PCOS.

Our study is an untargeted metabonomic study based on LC–MS. Altogether, 107 distinct metabolites were enriched. We discovered through pathway analysis that the different metabolites were mostly prevalent in the processes of beta-oxidation and mitochondrial beta-oxidation, among others, which are strongly related to PCOS (**Figures 3E, F**). Consequently, we verified that the distinct metabolites may have significant roles in PCOS. The main classes of differential metabolites include amino acids and peptides. According to prior research, considerable deviation in the plasma levels of amino acids was found in patients with PCOS (26). Furthermore, L-tyrosine and L-leucine have been shown to restore a regular menstrual cycle and ovulation in PCOS rat models (27). Hence, amino acids such as DL-tryptophan, L-lysine, L-histidine, L-tyrosine, L-phenylalanine, and isoleucine were chosen as prospective candidate metabolites to investigate their biological roles. The ROC analysis revealed that candidate amino acids showed a high PCOS diagnostic ability. This suggested that the amino acids may have diagnostic capacity in PCOS.

Most follicle somatic cells are granulosa cells (39, 40). Growing evidence suggests that functional alterations largely influence the growth, development, and maturation of follicles in granulosa cells (41, 42). Studies have extensively screened the differentially expressed genes in the granulosa cells to investigate the regulatory mechanisms governing the development of PCOS, with significant enrichment in pathways associated with metabolism, steroidogenesis, inflammation, cell proliferation, and apoptosis (43). Excessive apoptosis and delays in the proliferation of granulosa cells can lead to numerous cystic dilated follicles and atretic follicles in the ovarian cortex (44). The ovaries eventually exhibit polycystic alterations in the absence of mature follicles and corpus luteum development (45). Therefore, the malfunction of granulosa cells may account for abnormal folliculogenesis seen in PCOS (46). Herein, we demonstrate that isoleucine has a therapeutic role in the proliferation/apoptosis process, mitochondrial function, and oxidative stress in granulosa cells. Isoleucine, an essential branched-chain amino acid with anomalous content, is directly correlated with the development of PCOS (10, 47, 48). However, it remains to be ascertained how isoleucine affects PCOS. Our GC–MS/MS-targeted analysis revealed that isoleucine was significantly decreased in PCOS, suggesting that isoleucine was the potential target for PCOS treatment.

Furthermore, we tested hormone levels in cell culture supernatant. Important steroid hormones including P_4_ and T_0_ are produced by granulosa cells in developing follicles. They aid in the formation of ovarian follicles; however, an excess of androgen causes the granulosa cells to undergo autophagy and death, which negatively impacts ovarian function (49). Additionally, low levels of P_4_ can extend the life of dominant follicles (50), whereas high levels of P_4_ may eventually lead to dominant follicle atresia (43). This study shows that in comparison with the DHEA group, the levels of T_0_ and P_4_ were significantly decreased by the addition of isoleucine. These results indicated that isoleucine may participate in regulating the production of hormones.

When interpreting our findings, it is crucial to consider the limitations of our research. Samples of AT are difficult to obtain. Therefore, this study’s limitation was its limited sample size. Also, the need for more research in the relationship of high androgen and isoleucine is required. We hypothesized that isoleucine might regulate granulosa cell activity, including cell proliferation/apoptosis, sex hormone secretion, and mitochondrial malfunction of ovarian granulosa cells in PCOS induced by DHEA. Our study provides evidence that isoleucine is a promising candidate for PCOS therapy. However, more *in vivo* and *in vitro* investigations are required to learn more about the precise mechanism.

## Data availability statement

The original contributions presented in the study are included in the article/supplementary material. Further inquiries can be directed to the corresponding authors.

## Ethics statement

The studies involving human participants were reviewed and approved by Shanghai Tongren Hospital, Shanghai Jiao Tong University School of Medicine. The patients/participants provided their written informed consent to participate in this study.

## Author contributions

FD and LS designed the research. FD, LY, and DG performed the experiments. FD, JQ, and RZ analyzed the data. FD, RZ, and LS wrote the article. All authors contributed to manuscript revision, read, and approved the submitted version.
